# A Systematic Review of Lower Limb Strength Tests Used in Elite Basketball

**DOI:** 10.3390/sports12090262

**Published:** 2024-09-20

**Authors:** Tom Faulks, Pierpaolo Sansone, Sibi Walter

**Affiliations:** 1 Faculty of Health, University of Canterbury, Christchurch 8041, New Zealand; 2 Department of Movement, Human and Health Sciences, University of Rome “Foro Italico”, 00135 Rome, Italy; 3 Research Center for High Performance Sport, UCAM Universidad Católica de Murcia, 30107 Murcia, Spain

**Keywords:** basketball, lower limb strength testing, fitness testing, strength test

## Abstract

Background: Basketball players rely on their lower limb strength for speed and agility. Therefore, it is important for strength and conditioning coaches to seek methods to assess and develop lower limb strength. Objectives: This study aimed to identify tests and variables used to assess lower body strength among elite basketball players and to provide normative values for the commonly used strength tests. Methods: A review of PubMed, MEDLINE, Scopus, and SPORTDiscus was performed following the Preferred Reporting Items for Systematic Reviews and Meta-Analysis guidelines. The risk of bias was assessed using the Joanna Briggs Institute cross-sectional and cohort checklists. Results: Among the twelve reviewed studies, seven strength tests and five outcome variables were used. The most frequently used lower limb strength tests were the back squat (nine studies) and isometric mid-thigh pull (IMTP) (three studies), both reporting one repetition maximum (1RM) and peak force metrics. The most frequently used lower limb strength test was the back squat among males and IMTP among females. Conclusions: Among elite basketball players, the back squat 1RM is the most used lower limb strength test. However, across studies, a large variability was evidenced, which suggests that lower limb testing procedures are heterogeneous in this population.

## 1. Introduction

Basketball is a team sport involving speed and agility. The ability to accelerate, decelerate, jump, and change direction at different intensities and distances is critical for performance [1,2]. Elite basketball players execute more total match movements at high intensities, imposing greater physical demands on their lower limbs in comparison to less competitive levels of the sport [3,4,5]. To match these demands, elite basketball players are required to have considerable neuromuscular capacities to perform agile explosive movements, as well as high aerobic and anaerobic capacities to cope with the metabolic demands of the game [2,4,5,6,7,8,9].

As muscle power is a function of force production and velocity, well-developed lower limb maximal strength might be advantageous for basketball players [6]. During competitions, players must exert force in a rapid coordinated manner during the braking (eccentric), amortization (isometric), and propulsive (concentric) phases of movement while maintaining posture and position [10,11,12]. Furthermore, the game is characterized by frequent positional effort (i.e., picking, rebounding), where lower limb strength facilitates players in contesting to obtain a stable position on the court [5,13]. Therefore, greater maximal strength may underpin performance in a range of basketball-specific motor tasks that require large amounts of force production to rapidly move the body in the intended direction [4,10]. Research suggests that stronger athletes generally demonstrate superior force–time characteristics and, consequently, display better jumping, sprinting, and change of direction performance [14]. Several studies also support that maximal lower limb strength may positively influence sprinting speed, vertical jump, and change in direction performance [6,7,11,12,15,16,17]. At the collegiate level, lower limb strength has been related to future success in men’s basketball, with stronger players reaching higher levels of professional play than weaker counterparts [18]. Additionally, research has also shown that players who possess greater lower limb strength are awarded more playing time [16].

Considering the importance of lower limb strength in basketball, objective measures to target and develop strength training strategies are essential. Previously, various tests have been used to assess lower limb strength using dynamic, isometric, eccentric, and concentric muscle actions [19]. Each contraction type has been identified as critical to change in direction, sprinting, and jumping performance [6,7,10,11,12,14,20,21,22], and is therefore considered critical for on-court performance. In addition, the validity and reliability of the tests also warrant further consideration [14,18,23]. Indeed, a variety of testing methods can make it challenging for practitioners to select the most appropriate strength tests [23]. Currently, no reviews have identified the most used lower limb strength tests while highlighting the strength characteristics between different sex and age groups. Therefore, this review sought to identify the tests and variables used to assess lower limb strength characteristics in elite basketball players and to provide normative values for those strength tests. 

## 2. Materials and Methods

### 2.1. Study Design

This review followed the Preferred Reporting Items for Systematic Reviews and Meta-Analysis guidelines [24,25] and was registered with PROSPERO (CRD42023433235).

### 2.2. Search Strategy

The databases of PubMed, MEDLINE, Scopus, and SPORTDiscus were searched for relevant articles published prior to 30 November 2023. The following search string was used: (male OR female OR men OR women) AND (elite OR professional OR “division I” OR “division 1”) AND basketball AND (strength OR “strength testing” OR “performance testing” OR testing OR “physical characteristics” OR “physical qualities”). Reference lists of included studies were manually scanned for additional relevant studies. 

### 2.3. Eligibility Criteria

Studies included needed to (1) have full-text, peer-reviewed versions available in English; (2) use elite basketball players; (3) provide the strength test data for the lower limbs; and (4) clearly explain the testing methods and procedures. No restrictions were placed on the publication date. Elite basketball players were defined as players competing in professional leagues, Division I collegiate, and adolescent players who play in national competitions [1].

### 2.4. Study Selection

**Figure 1:**
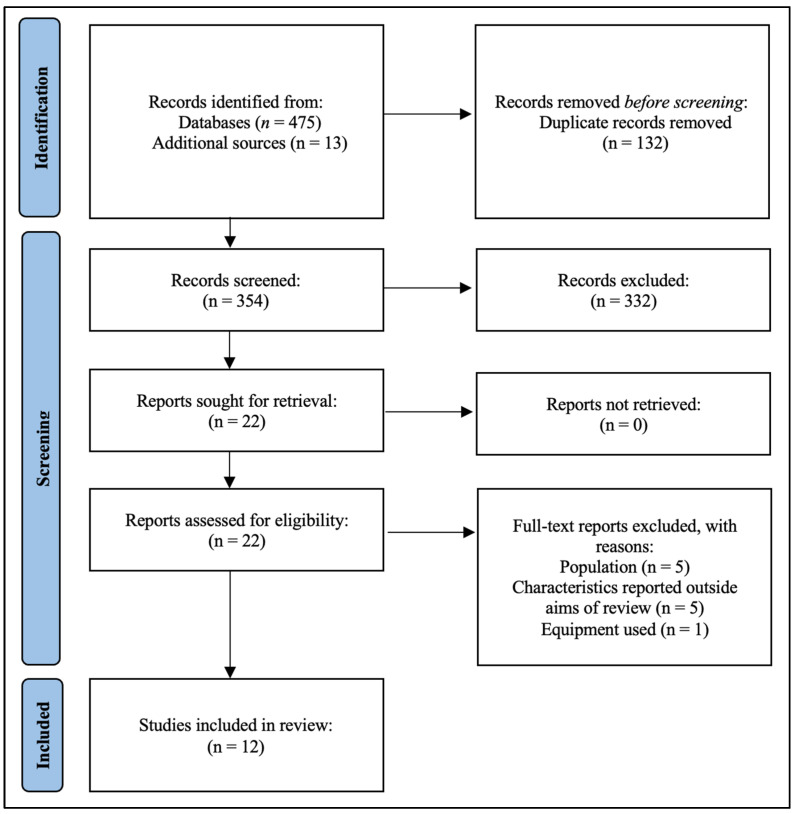
PRISMA flowchart showing the selection process of the reviewed studies.

The selection of the studies was a three-stage process. First, citations were independently identified for inclusion in the database search. Second, after all the duplicates were removed, relevant studies were sought for full-text versions, read independently by two authors, and evaluated for inclusion. Finally, eligible full-text studies were included in the review. The study selection flow is shown in Figure 1.

### 2.5. Assessment of Reporting Quality

The methodological quality of each study that met the inclusion criteria was subjected to the Joanna Briggs Institute (JBI) cross-sectional and cohort checklists [26]. The assessment was performed independently by two authors for the included studies in which cross-sectional and cohort design studies were involved. Studies were downgraded if there were issues or unclear aspects with the lower limb test, reliability and validity, statistical analysis, and study characteristics and methodology. Study quality was identified based on a previous systematic review [27], in which studies with a JBI score higher than 70% were classified as high quality, those scoring between 50 and 70% as medium quality, and those scoring >50% as low quality. 

### 2.6. Data Extraction

**Figure 2:**
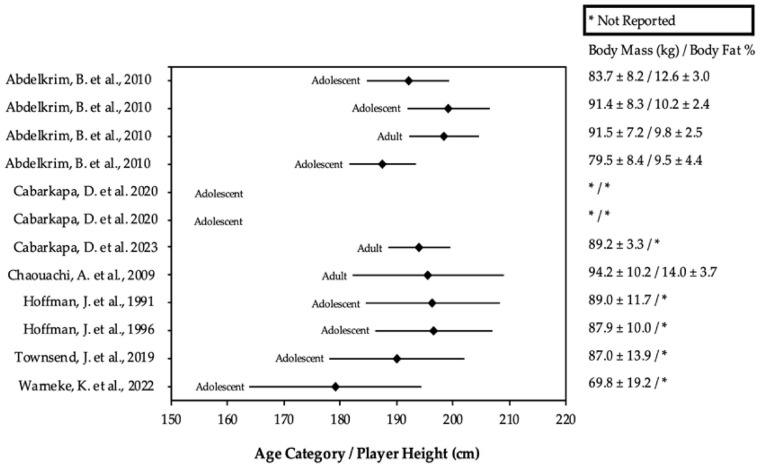
Anthropometric data of males according to age category. References: [7,8,16,17,18,30,31,32,34].

**Figure 3:**
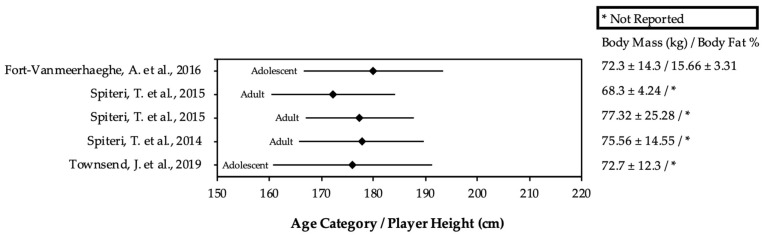
Anthropometric data of females according to age category. References: [11,12,16,33].

From the included studies, participant’s anthropometric and lower limb strength test data were extracted, which are presented in Figures 2 and 3 and Table 5. Such data were extracted from each study using the raw values provided. In the instance of an intervention study, only baseline measurements were used [28]. If multiple groups were included in the study, the control group was recorded to mitigate the effects of reporting biases [23]. 

### 2.7. Categorization and Presentation of Findings

Lower limb strength tests denoted in each study were categorized as back squat, eccentric box squat, concentric box squat, front squat, barbell deadlift, leg press, and isometric mid-thigh pull (IMTP). Because most studies reported derivatives of the back squat, we categorized all back squat variations that evaluated maximal dynamic strength as back squat to reduce the variability of strength tests used among elite basketball players. Studies were also stratified by age group and sex. Pre-defined age groups, based on the long-term athletic development framework [29], were (1) adulthood (>21 years) and (2) adolescence (12–21 years). 

## 3. Results

### 3.1. Selection of Studies

Following the deletion of duplicates, the literature search yielded a total of 354 studies. Of these, 332 studies were excluded based on the title and abstract screening. The full texts of 22 articles were retrieved, and the selection criteria were applied. After applying the inclusion criteria, 12 studies were finally included for review, as shown in Figure 1.

### 3.2. Assessment of Reporting Quality

Most studies (9/12) attained a high (70%) quality score, indicating a low risk of bias according to the JBI checklists. In 83% of studies, the validity and reliability of outcomes were a source of bias, while criteria for inclusion were observed as a source of bias in 33% of studies. All other sources of bias were commonly judged as low risk: setting described in detail (100%), control of confounding factors (92%), and appropriate statistical analysis (100%). For 10/12 reviewed studies, the validity and reliability of the exposure (question 3) were not applicable because no interventions were conducted. Similarly, strategies to deal with incomplete follow-ups (question 10) did not apply to the included cohort studies. Table 1 and Table 2 summarize the reporting quality scores across the included studies.

### 3.3. Overview of Included Study Characteristics

The final analysis included 192 male and 48 female elite basketball players, comprising 158 adolescents and 82 adults. Across the reviewed studies, lower limb strength characteristics were assessed using seven tests and five outcome variables, as summarized in Table 3 and Table 4.

### 3.4. Anthropometric Characteristics

Anthropometric data of the players were reported in 92% (11/12) of the reviewed studies. Male player heights ranged between 178 and 199 cm, while the heights of females ranged between 172 and 180 cm. Adolescent player heights ranged between 178 and 199 cm, while the heights of adults ranged between 172 and 198 cm. Male players weighed between 70 and 94 kg, while females weighed between 68 and 77 kg. Body composition was assessed in 33% (4/12) studies [7,30,31,32]. All data pertaining to the body composition are provided in Figure 2 and Figure 3. 

### 3.5. Lower Limb Strength Tests

1. Back Squat

The back squat was performed with the barbell positioned just above the acromion and required athletes to descend to 90° knee flexion or parallel position, which was attained when the greater trochanter reached the same level as the knee. Back squat performance was observed in nine studies [7,8,11,12,15,18,30,33,34] with absolute and relative 1RM strength as the outcome variables. The mean 1RM back squat loads ranged between 67 and 202 kg, while the mean relative back squat strength ranged from 0.95 to 1.09 kg • body weight (BW). Only absolute back squat strength was reported in males, while only relative strength was reported in females. The mean absolute strength ranged between 100 and 202 kg for males [7,8,15,18,30,33,34], and relative strength was between 0.95 and 1.09 kg • BW for females [11,12]. No studies measured back squat data performance in adolescent females.

2. Isometric Mid-Thigh Pull

The IMTP test protocol required maximal vertical isometric force application into the force plates for the requisite time in a static position. IMTP performance was observed in three studies [11,12,16], with the absolute peak force and peak force relative to body weight as outcome variables. The mean IMTP peak force ranged from 1248 to 2534 N, while the peak force relative to body mass was between 0.99 and 1.45 N • BW. The IMTP peak force was only reported in one study in adolescent males (2534 N) and females (1248 N) [16], while the relative IMTP peak force was only reported among adult females (0.99–1.45 N • BW) [11,12]. No studies measured IMTP performance among adult males.

3. Front Squat

The front squat was performed with the bar positioned across the anterior deltoids and required athletes to descend to a 90° knee flexion and immediately rise to an upright, standing position. Front squat performance was only reported in adolescent male and female players in two studies with 1RM [16] and 3RM protocols [17]. The mean front squat 1RM strength ranged between 84 and 126 kg, which was distinct from the 3RM load reported (66 kg). Adolescent males typically squatted greater 1RM front squat loads (66–126 kg) [16,17] than adolescent females (84 kg) [16], which was also greater than the 3RM load reported among adolescent males (66 kg) [17]. No studies measured front squat performance in adult male or female players.

4. Eccentric Squat

Eccentric squat strength was assessed by moving the knee joint to 90° flexion, whereby athletes were required to maintain a 3 s eccentric tempo. The bar position was the same as the back squat. Eccentric squat performance was only reported in two studies with kilograms lifted relative to body weight as the outcome variable, which was observed in adult female players [11,12]. The mean eccentric squat 1RM ranged between 1.14 and 1.44 kg • BW [11,12]. No studies measured eccentric squat performance in adolescent or adult males or adolescent females.

5. Concentric Squat

The concentric squat test protocol required athletes to begin seated on a box, with 90° knee flexion, and then ascend into a standing position with a 3 s concentric tempo. The bar position was the same as the back squat. Concentric squat performance was only reported in two studies with kilograms lifted relative to body weight as the outcome variable, which was observed in adult females [11,12]. The mean concentric squat 1RM ranged between 0.86 and 1.03 kg • BW. No studies measured concentric squat performance in adolescent or adult males or adolescent females.

6. Barbell Deadlift

The barbell deadlift test required athletes to grasp the barbell outside of their shin with extended elbows and then raise the barbell to a standing position through hip and knee extension. Barbell deadlift performance was only reported in one study [17], among adolescent males using a 3RM protocol. The mean barbell deadlift 3RM load reported was 88 kg [17].

7. Leg Press

The leg press test used an exhaustive submaximal protocol where the load was progressively increased until movement technique deteriorated, with athletes performing sets of 10 or fewer repetitions. Then, the 1RM strength was estimated using the Brzycki formula [32]. Leg press performance was only reported in one study [32], among adolescent females. The mean leg press 1RM load reported was 143 kg [32]. 

Lower limb strength was predominantly assessed using repetition maximum values, with 83% (10/12) studies using repetition maximum (1RM and 3RM), as shown in Table 4 and Table 5. The most frequent repetition max strength test used was the back squat (nine studies) [7,8,11,12,18,30,31,33,34].

**Table 4:** Mean data for reviewed studies.

Strength Characteristic	Test	Population	Mean Data	Citation Count
Isometric	IMTP	Adolescent male	2534 N	1
		Adolescent female	1248 N	1
		Adult female	1.26 N • BW	2
Dynamic	1RM back squat	Adult male	148 kg	3
		Adolescent male	151 kg	5
		Adult female	1.03 kg • BW	2
	1RM front squat	Adolescent male	126 kg	1
		Adolescent female	84 kg	1
	3RM front squat	Adolescent male	66 kg	1
	1RM leg press	Adolescent female	143 kg	1
Eccentric	1RM eccentric squat	Adult female	1.31 kg • BW	2
Concentric	1RM concentric squat	Adult female	0.95 kg • BW	2
	3RM barbell deadlift	Adolescent male	88 kg	1

Notes: IMTP, Isometric mid-thigh pull; RM, Repetition maximum; BW, Body weight; N, Newtons; KG, Kilograms.

## 4. Discussion

Lower limb strength is an essential physical capacity necessary for basketball players to perform sport-specific actions such as jumps, changes in direction, and static efforts, which happen frequently during gameplay. In order to design effective lower limb strength training strategies, the objective testing of lower limb strength capacity and normative values are needed. Currently, there are numerous lower limb strength tests, making it challenging for a practitioner to select the most appropriate strength test. Therefore, this systematic review identified the commonly used lower limb strength tests, variables, and characteristics in elite basketball players and highlighted their normative values. 

### 4.1. Tests and Outcome Variables

Anthropometric data were collected using low-cost, easy-to-implement tests, such as electronic scales, stadiometers, and skin fold assessments. Lower limb strength was most assessed using the back squat [7,8,11,12,18,30,31,33,34] and IMTP [11,12,16] reporting the RM and peak force metrics. Of those, the back squat was most frequently assessed using 1RM protocols, yielding absolute and relative strength measures as outcome variables. However, the back squat 1RM protocol was primarily reported in male players (78% of back squat studies) [7,8,18,30,31,33,34]. Among females, the IMTP was commonly used to measure their lower limb strength [11,12,16]. 

This review highlights the large variability in the tests used to assess lower limb strength among elite basketball players. Interpreting test results is further complicated by multiple methodologies and procedures to measure multiple outcome variables. For instance, poorly reported variables such as the squat depth and movement velocity can influence the maximum force produced, because the force produced is dependent on the muscle shortening velocity and its length [35,36,37,38,39,40,41]. Furthermore, the validity and reliability of commonly used tests were often not reported (Table 4 and Table 5). As each test and testing methodology has an inherent level of measurement fidelity, the nuances associated with various procedures may influence the results and need to be considered, especially when comparing results between studies. Therefore, researchers, governing bodies, and practitioners are encouraged to work collaboratively to standardize the lower limb strength tests that are most appropriate for elite basketball players. This will help establish meaningful normative data.

### 4.2. Anthropometry and Body Composition

Height measured at the National Basketball Association draft combine has been identified as a predictor of future performance [42] and hence should be part of a standardized anthropometrical assessment. This may be explained by the constraint placed by the basket, set at a 3.05 m height, which favors taller players in both offensive and defensive scenarios. As the execution of these skills impacts winning or losing, it seemingly follows that basketball inherently favors taller players, as reflected by anthropometry characteristics reported across the studies in this review [28,43]. However, concerning the relevance to lower limb strength, physics principles, including the impulse–momentum relationship and inertia, would suggest that body mass is important for basketball performance [14]. Basketball strength coaches, therefore, would benefit by recording body mass to provide insight into the strength-to-mass ratio, which reflects on an individual’s ability to exploit their levels of strength relative to their body mass.

A lower body fat percentage means higher fat-free mass, which is advantageous for the strength-to-mass ratio, influencing players’ relative power and mechanical efficiency [42,44]. Thus, body fat percentage could certainly be considered as a specific performance factor where increases in maximal strength without the accretion of body fat are desirable [44]. In fact, a previous review by Sansone et al. [28] found that body fat differentiates competitive levels in basketball, with international-level players having lower body fat than national and regional-level players. In this review, body composition differed between sex and age. Specifically, male adults and adolescents possessed lower body fat percentages than female players [28]. When body fat percentage was observed between males, adult players (13–14%) had higher fat mass than male adolescent players (10%). However, the comparison of body fat percentages across the studies included in this review should be made with caution as the anatomical landmarks and equations used were not always consistent, leading to varying levels of accuracy in body fat estimates.

### 4.3. Lower Limb Strength Tests

1. Back Squat

The basketball game demands well-developed lower limb muscle strength to perform intense, multidirectional movements [7,8,10,11,12,14,17,18,20,30,33,45]. Consequently, practitioners extensively use the back squat exercise to assess and develop lower limb muscle strength [7,8,14,15,16,18,20,31,34]. This review suggests that the back squat test’s absolute strength was frequently used to report maximal strength capabilities, which may be due to the practicality and simplicity of testing. The highest back squat loads reported in the literature were from the Tunisian National Team male basketball players (adolescents—183.3 ± 17.8 kgs, adults—201.5 ± 16.2 kgs) 30. Adult males (100–202 kg) tended to record comparable 1RM back squat loads to adolescent males (119–183 kg), but no studies reported absolute 1RM back squat loads among females to draw conclusions. However, relative strength was reported in adult females (0.95–1.09 kg • BW), with no studies available across adolescent females or males to draw conclusions regarding the relative back squat strength. Considering the varying abilities of players, it is important to consider that different testing protocols (i.e., 90° knee flexion versus femur parallel with the floor) have been used across studies. Thus, the different methods used to quantify back squat strength limit comparisons. Additionally, basketball strength coaches would benefit from reporting outcome variables representing the absolute and relative strength to help establish normative data that can assist in assessing basketball players’ lower limb strength. Therefore, the largely consistent usage of the back squat exercise in elite basketball settings suggests that this test is favored by researchers and practitioners working with elite male basketball players.

2. Isometric Mid-Thigh Pull

The IMTP is an efficient isometric strength test to analyze force–time characteristics, such as the rate of force development (RFD) and impulse, while avoiding excessive mechanical loading [4,6,14]. Given that RFD and impulse are determinants for fast, explosive movements limited by the time frame for force application, it is surprising that limited evidence exists pertaining to the IMTP in elite basketball players. Nonetheless, such evidence suggests that a broad range of relative peak force exists among adult female players (0.99–1.45 N • BW) [11,12]. Thus, the ability to draw conclusions is hindered by the insufficient data reported regarding sex and age groups. Further research is required to investigate isometric strength characteristics in elite basketball players to explore whether differences in peak force and temporal metrics (RFD and impulse) are apparent between age groups according to sex. Such exploration may also provide sufficient evidence to help identify the normative values of IMTP in elite basketball players.

3. Front Squat

The front squat allows athletes to perform the squatting movement with a more upright posture in comparison to the back squat, reducing stress on the lumbar spine and knee joint [37,46]. Despite this, when observing front squat performance across sexes and age groups, we noted insufficient data and high heterogeneity of testing methods, which limits our possibilities to draw firm conclusions. Of the two studies that reported the front squat, one reported 1RM loads 16, and the other reported 3RM loads [17]. Interestingly, front squat performance was only reported among adolescent players (male 1RM: 66–126 kg, female 1RM: 84 kg). The 1RM loads attained during the front squat were consistently lower than the back squat, which is in line with previous research [46]. These differences are likely due to the inherent change in bar position that influences the joint dominancy strategy and the ability to produce external force [35,46,47]. The limited evidence and heterogenous testing methods are insufficient to draw conclusions regarding age groups and sex.

4. Eccentric Squat

The eccentric squat test reveals useful information regarding the stretch-shortening cycle and assessing this is critical for basketball-specific decelerating, changing direction, and jumping performance [3,5,11,31,48,49,50,51,52]. Further, the research underpinning eccentric strength training is vast and appears to be fostering increased interest among practitioners, with data suggesting potential applications in basketball players to develop deceleration capacities required in rapid eccentric braking movements [48,49,50,51,53,54,55]. However, eccentric squat 1RM strength (1.14–1.44 kg • BW) was only reported in two studies using adult female players [11,12]. A possible reason for the lack of research exploring the eccentric squat might be that assessing each muscle action individually is considered more time-consuming while providing highly interrelated information to a back squat test [56]. Considering the insufficient data investigating eccentric strength qualities in elite basketball players and their importance for basketball-specific movements, further research is needed to explore potential differences and develop normative values across sex and age groups.

5. Concentric Squat

The concentric squat strength test reflects the force-producing capability of muscle during the propulsive phase of a movement. Two studies reported the concentric box squat 1RM (0.86–1.03 kg • BW), both among adult females [11,12]. It would be advantageous to collate further research to fully understand the concentric strength standards for each sex and age group. Notably, the 1RM loads attained during the concentric squat were consistently lower than those during the eccentric squat. This difference could be due to the relationship between contraction velocity and the force-producing capabilities of muscle. When compared directly, eccentric muscle actions can produce force in amounts typically 20–60% greater than during concentric activities [48,51,53]. It is important to note that factors such as the force–length relationship, neural factors, and training history also contribute to the force a muscle can exert [35,36,39].

6. Barbell Deadlift

The barbell deadlift is a concentric lower limb strength exercise requiring large muscular forces in a hip extension motion. Such hip extensor force capacity has been identified as crucial for lateral shuffling and sprinting performance [5,57,58,59]. Only one study reported mean barbell deadlift performance using a 3RM protocol in adolescent males [17]. Thus, the current findings indicate that more research is required to provide basketball researchers and practitioners with sufficient information to confidently evaluate deadlift performance in elite basketball players. It is surprising that, currently, there is no evidence to support the hex bar deadlift test, given [60] the biomechanical similarity of the starting position of a hex bar deadlift to the basketball base stance.

7. Leg Press

The leg press is a machine-based dynamic lower limb strength test targeting the knee and hip extensors [61,62]. The leg press action utilizes the same muscles and joint angles as a back squat [61,62]. However, the leg press may not offer basketball strength coaches relevant insights, because it does not involve the coordinated interaction of the trunk and lower limbs to stabilize and support explosive jumping and sprinting movements [37,61,63]. This is likely why only one study utilized this testing method in adolescent female players [32]. In female players, the current review suggests that adolescents exhibit greater 1RM strength on the leg press than the front squat, possibly because of the reduced stabilization requirements and motor complexity, permitting more force to be applied in a linear path on the leg press [61].

### 4.4. Limitations

While the current review presents a comprehensive description of lower limb strength tests, variables, and data reported in elite basketball settings, some limitations should be considered. Firstly, limiting the database search to English articles likely ignores key data published in other languages and thus introduces reporting bias. Secondly, the lack of female-specific basketball studies and the smaller sample size used in existing female-specific basketball studies make it difficult to conclude the normative lower limb strength levels for females. Finally, as seen in Table 5, the varying testing methodologies and validity and reliability statistics found in the literature make establishing normative data challenging. 

### 4.5. Practical Application

Currently, the lack of similar testing methods, resources, and normative data makes it challenging to establish a standardized strength testing protocol and compare physical performance measures. However, the summary of strength tests, testing methods, and normative data provided in this review will benefit physical performance coaches in implementing a reproducible strength testing protocol. The use of a 1–3RM back squat test and recording the relative strength is proposed as a standard testing protocol for basketball players if they are experienced in resistance training and possess movement competency. Alternatively, the use of the IMTP for measuring lower limb strength is recommended for players who do not have resistance training experience and squat movement proficiency. 

## 5. Conclusions

This review provides a comprehensive summary of lower limb strength tests for physical performance coaches working in basketball. While the variability in testing protocols and assessed measures is apparent, the back squat test and the 1RM assessment method are the most used. Considering the variability in studies reporting absolute vs. relative strength, the authors recommend that practitioners and researchers record both the absolute and relative strength so that the testing is specific to an athlete’s resistance training experience, age, and sex. Physical performance coaches working with athletes with less or no resistance training experience could use the IMTP instead of the back squat test. 

## Figures and Tables

**Table 1:** Summary of Joanna Briggs Institute critical appraisal for cross-sectional studies.

Study	Question								Total
	1	2	3	4	5	6	7	8	
Abdelkrim et al., 2010 [30]	N	Y	NA	Y	Y	Y	Y	Y	75%
Abdelkrim et al., 2010 [31]	N	Y	NA	Y	Y	Y	Y	Y	75%
Cabarkapa et al., 2023 [32]	N	N	NA	Y	Y	Y	N	Y	50%
Chaouachi et al., 2009 [7]	N	Y	NA	Y	Y	Y	Y	Y	63%
Fort-Vanmeerhaeghe et al., 2016 [33]	Y	Y	NA	Y	N	N	N	Y	50%
Spiteri et al., 2014 [12]	Y	Y	NA	Y	Y	Y	N	Y	75%
Spiteri et al., 2015 [11]	Y	Y	NA	Y	Y	Y	N	Y	75%
Townsend et al., 2019 [16]	N	Y	NA	Y	Y	Y	N	Y	75%
Warneke et al., 2022 [17]	Y	Y	NA	Y	Y	Y	N	Y	75%
Overall									68%

Y, yes; N, no; NA, not applicable.

**Table 2:** Summary of Joanna Briggs Institute critical appraisal for cohort studies.

Study	Question											Total
	1	2	3	4	5	6	7	8	9	10	11	
Cabarkapa et al., 2020 [18]	Y	Y	NA	Y	Y	Y	N	Y	Y	NA	Y	73%
Hoffman et al., 1991 [8]	Y	Y	N	Y	Y	Y	N	Y	Y	NA	Y	73%
Hoffman et al., 1996 [34]	Y	Y	N	Y	Y	Y	N	Y	Y	NA	Y	73%
Overall												73%

Y, yes; N, no; NA, not applicable.

**Table 3:** Commonly used strength tests from the reviewed studies.

Strength Characteristic	Test	Number of Studies
Dynamic	Back squat	9/12
	Front squat	2/12
	Leg press	1/12
Isometric	Isometric mid-thigh pull	3/12
Concentric	Barbell deadlift	1/12
	Concentric box squat	2/12
Eccentric	Eccentric box squat	2/12

**Table 5:** Summary of lower limb strength test variables from the reviewed studies [7,8,11,12,16,17,30,31,32,33,34].

Study	Sex (*n*)	Age Category	Groups	Test	Results (Mean ± SD)
Abdelkrim et al., 2010 [30]	Male (*n =* 15)	Adolescents	Tunisian National Team	Back Squat 1RM (kg)	183.0 ± 24.0
	Male (*n =* 15)	Adolescents	Tunisian National Team	Back Squat 1RM (kg)	183.3 ± 17.8
	Male (*n =* 15)	Adults	Tunisian National Team	Back Squat 1RM (kg)	201.5 ± 16.2
Abdelkrim et al., 2010 [31]	Male (*n =* 18)	Adolescents	Tunisian National Team	Back Squat 1RM (kg)	128.1 ± 11.9
Cabarkapa et al., 2020 [18]	Male (*n =* 10)	Adolescents	NCAA D1	Back Squat 1RM (kg)	153.3 ± 26.2
	Male (*n =* 8)	Adolescents	NCAA D1	Back Squat 1RM (kg)	144.6 ± 23.8
Cabarkapa et al., 2023 [32]	Male (*n =* 6)	Adults	Professional Team	Back Squat 1RM (kg)	99.5 ± 12.8
Chaouachi et al., 2009 [7]	Male (*n =* 14)	Adults	Tunisian National Team	Back Squat 1RM (kg)	143.0 ± 13.4
Fort-Vanmeerhaeghe et al., 2016 [33]	Female (*n =* 9)	Adolescents	National Spanish Basketball Federation	Leg Press 1RM (kg)	143.0 ± 12.96
Hoffman et al., 1991 [8]	Male (*n =* 9)	Adolescents	NCAA D1	Back Squat 1RM (kg)	119.4 ± 25.2
Hoffman et al., 1996 [34]	Male (*n =* 14)	Adolescents	NCAA D1	Back Squat 1RM (kg)	143.4 ± 24.3
Spiteri et al., 2014 [12]	Female (*n =* 12)	Adults	Women’s National Basketball League	Back Squat 1RM (kg • BW)	1.04 ± 0.04
	Female (*n =* 12)	Adults	Women’s National Basketball League	Concentric Squat 1RM (kg • BW)	0.96 ± 0.23
	Female (*n =* 12)	Adults	Women’s National Basketball League	Eccentric Squat 1RM (kg • BW)	1.34 ± 0.34
	Female (*n =* 12)	Adults	Women’s National Basketball League	IMTP (N • BW)	1.35 ± 0.38
Spiteri et al., 2015 [11]	Female (*n =* 6)	Adults	Women’s National Basketball League	Back Squat 1RM (kg • BW)	1.09 ± 0.32
	Female (*n =* 6)	Adults	Women’s National Basketball League	Concentric Squat 1RM (kg • BW)	1.03 ± 0.32
	Female (*n =* 6)	Adults	Women’s National Basketball League	Eccentric Squat 1RM (kg • BW)	1.44 ± 0.20
	Female (*n =* 6)	Adults	Women’s National Basketball League	IMTP (N • BW)	1.45 ± 0.37
	Female (*n =* 6)	Adults	Women’s National Basketball League	Back Squat 1RM (kg • BW)	0.95 ± 0.17
	Female (*n =* 6)	Adults	Women’s National Basketball League	Concentric Squat 1RM (kg • BW)	0.86 ± 0.18
	Female (*n =* 6)	Adults	Women’s National Basketball League	Eccentric Squat 1RM (kg • BW)	1.14 ± 0.22
	Female (*n =* 6)	Adults	Women’s National Basketball League	IMTP (N • BW)	0.99 ± 0.13
Townsend et al., 2019 [16]	Male (*n =* 8)	Adolescents	NCAA D1	Front Squat 1RM (kg)	126.1 ± 17.7
	Male (*n =* 8)	Adolescents	NCAA D1	IMTP (N)	2534.1 ± 368.0
	Female (*n =* 15)	Adolescents	NCAA D1	Front Squat 1RM (kg)	83.6 ± 12.5
	Female (*n =* 15)	Adolescents	NCAA D1	IMTP (N)	1248.0 ± 377.2
Warneke et al., 2022 [17]	Male (*n =* 42)	Adolescents	German National Basketball League	Front Squat 3RM (kg)	66.01 ± 31.11
	Male (*n =* 42)	Adolescents	German National Basketball League	Barbell Deadlift 3RM (kg)	87.56 ± 43.16

Notes: D1, Division 1 competition; NCAA, National Collegiate Athletic Association; IMTP, Isometric mid-thigh pull; RM, Repetition maximum; BW, Body weight; N, Newtons; KG, Kilograms.

## Data Availability

The data that support the findings of this study are available upon request.
